# Use of recombinant human deoxyribonuclease in pediatric intensive care unit – a single-center experience

**DOI:** 10.1590/1984-0462/2022/40/2020169

**Published:** 2021-09-01

**Authors:** Daniel Meireles, Sofia Ribeiro Fernandes, Alzira Sarmento, Telma Barbosa, Manuel Ferreira Magalhães, Ana Ramos, Paula Cristina Fernandes

**Affiliations:** a Centro Hospitalar Universitário do Porto, Porto, Portugal.

**Keywords:** Deoxyribonuclease I, Pulmonary atelectasis, Critical care, Ventilation, Desoxirribonuclease I, Atelectasia pulmonar, Cuidados críticos, Ventilação

## Abstract

**Objective:**

Dornase alfa (rhDNase) reduces the viscosity of purulent sputum in the lungs. The use in patients with cystic fibrosis (CF) is proven. However, the evidence of its applicability to other conditions is limited. This study aims to present the authors’ experience with the use of rhDNase in non-CF patients admitted to the Pediatric Intensive Care Unit (PICU). At the study center, rhDNase was used during flexible bronchoscopies in 24 cases, of which 20 (83%) had atelectasis and seven (29%) were admitted to PICU. Four patients (57%) were on invasive mechanical ventilation (MV).

**Case description:**

Two cases of daily rhDNase administration at PICU are presented: patient A was an 8-year-old boy admitted with septic shock and acute respiratory distress syndrome (ARDS). The patient required mechanical ventilation with aggressive settings and experienced several clinical complications. On D50, he started rhDNase treatment with an improvement in FiO_2_, PaCO_2_ and PaO_2_/FiO_2_ ratio according to radiologic findings. He was extubated on D23 of treatment.

Patient B was a 17-month-old girl admitted with a convulsive status epilepticus who experienced respiratory complications (infectious and barotrauma) with ARDS, requiring aggressive ventilation. She initiated rhDNase treatment on D60. During the treatment an improvement in FiO_2_, PaO_2_/FiO_2_ ratio and a tendency of PaCO_2_ decrease were found. She had radiological improvement. No complications were described.

**Comments:**

RhDNase may be a helpful and safe tool to use in PICU prolonged intubated patients with ventilator-induced lung injury. Further studies are needed to assess and propose valid indications.

## RESUMO

**Objetivo:**

A dornase alfa (rhDNase) reduz a viscosidade da expectoração pulmonar purulenta. O seu benefício em doentes com fibrose cística (FC) é comprovado, no entanto, as evidências da sua aplicabilidade em outras patologias são limitadas. Este estudo pretende apresentar a nossa experiência com a utilização de rhDNase em doentes não-FC internados na Unidade de Terapia Intensiva Pediátrica (UTIP). No nosso centro, utilizamos rhDNase em 24 casos, durante broncoscopia flexível. Desses, 20 (83%) apresentaram atelectasia e sete (29%) foram admitidos na UCIP. Quatro doentes (57%) estavam em ventilação mecânica (VM).

**Descrição do caso:**

Apresentamos dois casos de administração diária de rhDNase na UTIP: o paciente A tem 8 anos, sexo masculino, internado por choque séptico e síndrome da angústia respiratória aguda (SARA). Necessitou de VM com parâmetros agressivos e teve várias complicações clínicas. No D50 iniciou rhDNase com melhoria da FiO_2_, PaCO_2_ e relação PaO_2_/FiO_2_, assim como dos achados radiológicos. Foi extubado no D23 de tratamento. A paciente B é uma criança de 17 meses, sexo feminino, internada por estado de mal epilético, com necessidade de ventilação agressiva e com várias complicações respiratórias (infeciosas e de barotrauma). Iniciou rhDNase no D60. Ao longo do tratamento verificamos melhoria da FiO_2_ e da relação PaO_2_/FiO_2_, assim como uma tendência para a diminuição da PaCO_2_ e melhoria radiológica. Não foram descritas complicações para ambos os pacientes.

**Comentários:**

A rhDNase pode ser uma ferramenta útil e segura para pacientes em VM prolongada e com lesão pulmonar induzida pelo ventilador. São necessários estudos adicionais para avaliar e propor indicações válidas.

## INTRODUCTION

Dornase alfa (rhDNase) is a purified solution of recombinant human deoxyribonuclease (rhDNase) that reduces the viscosity of purulent sputum in the lungs through the digestion of airway extracellular deoxyribonucleic acid (DNA) released from neutrophils.^1^ ^,^ ^2^ This mechanism allows to improve the clearance of respiratory secretions. Nowadays, Pulmozyme^®^ (rhDNase) is licensed for adults and children aged five years and older in North America and Europe.^2^

Currently, the use of nebulized dornase alfa is well established in the treatment of patients with cystic fibrosis (CF), with improvement in mortality and morbidity when compared with placebo and other medications.^3^ The use of rhDNase in other lung diseases, such as asthma, bronchiolitis or primary ciliary dyskinesia, has been studied with variable degrees of success. But a systematic review published by Cochrane in 2012 that analyzed the administration of rhDNase in viral bronchiolitis in children younger than 24 months of age concluded that this intervention did not reduce the hospitalization length of stay or accelerate the clinical improvement.^1^

The evidence of its use in other diseases is limited. Riethmueller et al. evaluated the use of rhDNase in pediatric patients younger than two years of age recovering from surgery for congenital heart disease.^4^ The primary outcome was rate of reintubation and there was no difference between 0.9% saline (n=50) or dornase alfa (n=50). But in a *post hoc* analysis, the authors reported a shorter length of mechanical ventilation, shorter length of stay at the Intensive Care Unit (ICU), and lower cost of hospitalization in the rhDNase group.^4^ In 2009 the same author published a retrospective study comparing the endotracheal administration of dornase alfa *vs*. saline instillation in intubated patients, of the general Pediatric ICU (PICU), with atelectasis or lung infiltrates on chest x-ray.^5^ In this study, patients treated with rhDNase had better improvement in the atelectasis and quicker reduction of the fraction of inspired oxygen (FiO_2_) and peak inspiratory pressure.^5^

Other studies with smaller samples were performed to evaluate the efficacy of dornase alfa treatment. A retrospective study published in 2005 by Hendricks et al. evaluated the use of nebulized rhDNase in non-ventilated and instilled rdDNase in PICU intubated patients (n=30), and 57% of these patients showed improvements in at least two of the measured variables (respiratory rate, FiO_2_ and partial pressure of carbon dioxide [PaCO_2_]).^6^ Fedakar et al. published in 2012 a non-randomized study on neonatal ICU patients (n=22) and showed improvements in chest x-ray, respiratory rate, FiO_2_, and PaCO_2_ in 82% of the infants treated with nebulized rhDNase twice a day during three days.^7^ Other retrospective study published in 2009 by Prodhan et al. included pediatric cardiac ICU patients (n=38) and the results showed improvements in chest x-ray scores of atelectasis in patients treated with dornase alfa twice a day.^8^

The aim of the present study was to describe two cases from the authors’ experience with the daily treatment with dornase alfa in PICU, specifically in patients with prolonged invasive ventilation and with possible/probable ventilator-induced lung injury (VILI) such as recurrent atelectasis or lung infiltrates on chest x-ray.

In the hospital where the study was conducted, a total of 222 flexible bronchoscopies (FB) were performed between January 2013 and June 2019 (43 times for therapeutic purposes). In these patients, rhDNase was instilled in 24 FB and the most frequent indication was atelectasis (in 83% of therapeutic FB). In those therapeutic FB, several patients had a chronic disease and the neuromuscular disease was the most prevalent among them (42%). Seven of the 24 patients treated with rhDNase through FB (29%) were admitted to PICU. Two of these patients were treated with a first dose of dornase alfa through FB and daily maintained therapy by endotracheal tube instillation. The rhDNase was administrated twice a day in a dose of 2500 UI (every 12 hours).

The analyzed clinical data on patients included respiratory rate and ventilation parameters such as peak inspiratory pressure, FiO_2_, PaCO_2_ and the partial pressure of oxygen (PaO_2_)/FiO_2_ ratio. The data were descriptively analyzed in terms of the aforementioned measures.

In both cases, for the objective evolution assessment, each chest x-ray was checked for three items: atelectasis, hyperinflation and mediastinal shift. In order to standardize the chest x-ray interpretation, a scoring system for atelectasis previously used by Hendriks et al. was considered.^6^ For atelectasis, a partial atelectasis of one lobe had one point, and complete atelectasis had two points. The presence or absence of hyperinflation was scored for one point or zero points, respectively. The presence or absence of a mediastinal shift was scored as one or zero. These results were summed for each x-ray. When relevant, an exploratory inferential analysis was performed for comparing clinical data pre- and post-rhDNAse treatment.

## CASE REPORT

Patient A was an 8-year-old boy (Table 1) with a diagnosis of B-cell acute lymphoblastic leukaemia treated with chemotherapy regimen with vincristine, mercaptopurine and methotrexate. He was transferred from other hospital by septic shock secondary to pseudomembranous colitis. Even after the initiation and optimization of the antibiotic treatment, he had a poor clinical course and was admitted to PICU.

**Table 1:** Clinical characteristics of the patients.

Characteristic	Patient A	Patient B
Sex	Boy	Girl
Mechanical ventilation duration (days)	70	75
Length of stay at the Pediatric Intensive Care Unit (days)	86	94
Day of the start of dornase alfa treatment	50	60
Dornase alfa treatment duration (days)	23	17

At admission, the patient was hemodynamically unstable, on high doses of vasoactive amines. On the first day (D1) of admission, the patient developed an acute respiratory distress syndrome (ARDS) with a PaO_2_/FiO_2_ ratio of 132 and he initiated conventional mechanical ventilation with aggressive settings (pressure regulated volume control with peak inspiratory pressure 24 cmH_2_O, positive end-expiratory pressure 12 cmH_2_O and FiO_2_ 1).

During PICU stay, he maintained aggressive ventilator parameters (FiO_2_>75% and maximum peak of inspiratory pressure 36 cmH_2_O), leading to an optimization on ventilatory modes (high-frequency ventilation during D14-D19, though without any further improvement). After this, he experienced some clinical complications, such as recurrent atelectasis, and pneumothoraces treated with insertion of thoracic tubes and chemical pleurodesis.

**Figure 1:**
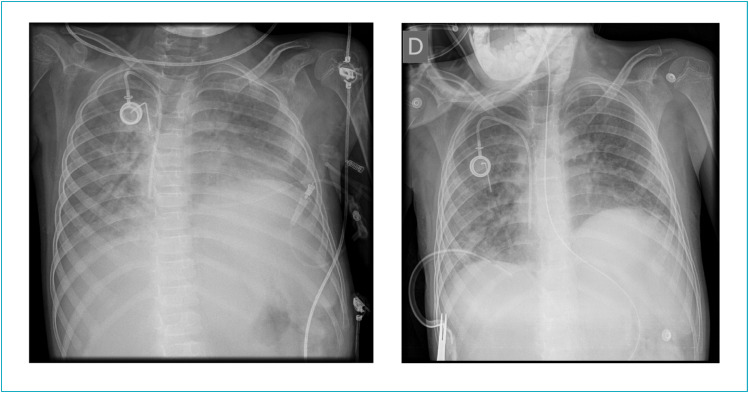
Chest x-ray performed on the first day and 24 hours after suspending dornase alfa therapy in patient A.

On D50, he started intratracheal rhDNase administration (Pulmozyme^®^), at a dosage of 2500 UI every 12 hours. Before the initiation of rhDNase, the x-ray had pulmonary atelectasis with a score of three (Figure 1A).

During the 23 days of dornase alfa treatment, an improvement in PaO_2_/FiO_2_ ratio and ventilatory parameters was verified such as FiO_2_, respiratory rate (RR, number of breaths per minute), PaCO_2_ and peak inspiratory pressure. These results are demonstrated in Table 2. Indeed, when performing an exploratory analysis of the median of FiO_2_, PaCO_2_ and PaO_2_/FiO_2_ ratio, a difference between the period before rhDNase and after the treatment initiation was found (Table 2). After the beginning of rhDNase treatment, the patient remained another 23 days in mechanical ventilation (he was already 50 days of intubation at the beginning of treatment), with favourable evolution. The chest x-ray 24 hours after the extubation day is presented in Figure 1B, with a score of one. The treatment with rhDNAse was stopped right after extubation (total treatment duration of 23 days). Then, the patient did not require mechanical ventilation again and he was discharged to a pediatric ward on the 86^th^ day of PICU stay.

**Table 2:** Clinical parameters before and after dornase alfa treatment.

	Patient A		Patient B	
	Before dornase alfa (IQR)	After dornase alfa (IQR)	Before dornase alfa (IQR)	After dornase alfa (IQR)
Respiratory rate (moments/minute)	38 (11.0)	32 (14.0)	35 (0.75)	28 (14.0)
Peak Inspiratory Pressure (mmHg)	26 (6.0)	19 (4.50)	21 (4.0)	22 (8.25)
Fraction of inspired oxygen (%)	95 (20.0)	35 (15.0)	100 (18.75)	50 (42.0)
Partial pressure of carbon dioxide (mmHg)	65 (20.0)	48 (10.50)	49 (11.50)	44 (15.0)
Partial pressure of oxygen/Fraction of inspired oxygen ratio	133 (55.0)	343 (209.0)	122 (44.75)	317 (368.50)

Values are expressed in median and interquartile range (IQR).

Patient B was a 17-month-old girl (Table 1) admitted to PICU with a generalized convulsive status epilepticus. She was intubated and initiated conventional mechanical ventilation at the admission day (D1) and in that time, she was hemodynamically unstable, needing vasoactive amines (which were stopped on D6).

During the PICU stay, she had several respiratory complications, mainly recurrent lung infection: D1 – adenovirus and metapneumovirus (nasopharyngeal aspirate); D24 – cytomegalovirus (bronchoalveolar lavage); D40 – metapneumovirus (nasopharyngeal aspirate); D65 – *Stenotrophomonas maltophila* (bronchial aspirate). The patient experienced other complications, such as pneumothorax and pneumomediastinum, on D14, with required thoracic tube insertion.

**Figure 2:**
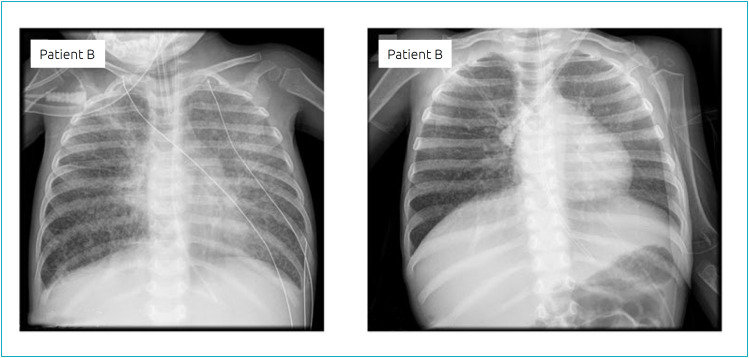
Chest x-ray performed on the first day and 24 hours after suspending dornase alfa therapy in patient B.

Indeed, she needed high ventilation parameters (30 cmH_2_O, maximum of peak inspiratory pressure) and developed an ARDS with a PaO_2_/FiO_2_ ratio under 150, despite the optimization of ventilatory parameters. The patient initiated intratracheal rhDNase treatment on D60, with daily administration (Pulmozyme^®^) of 2500 UI every12 hours. At this time, her chest x-ray had a score of three (Figure 2A).

Again, during the treatment with rhDNase, a favourable evolution of the respiratory condition was observed, specifically concerning FiO_2_, respiratory rate, PaCO_2_, and PaO_2_/FiO_2_ ratio and peak inspiratory pressure (Table 2). Moreover, a difference between the period before and after the treatment for FiO_2_, PaCO_2_ and PaO_2_/FiO_2_ ratio was verified. The median of PaCO_2_ decreased during the treatment (Table 2). The patient gradually improved since the beginning of rhDNAse treatment and she was extubated on D75 (15 days of treatment). The treatment was stopped at the extubation time, when the chest x-ray was normal – score of 0-1 (Figure 2B). Likewise, patient B did not require mechanical ventilation again and was discharged on the 94^th^ day of PICU stay.

**Figure 3:**
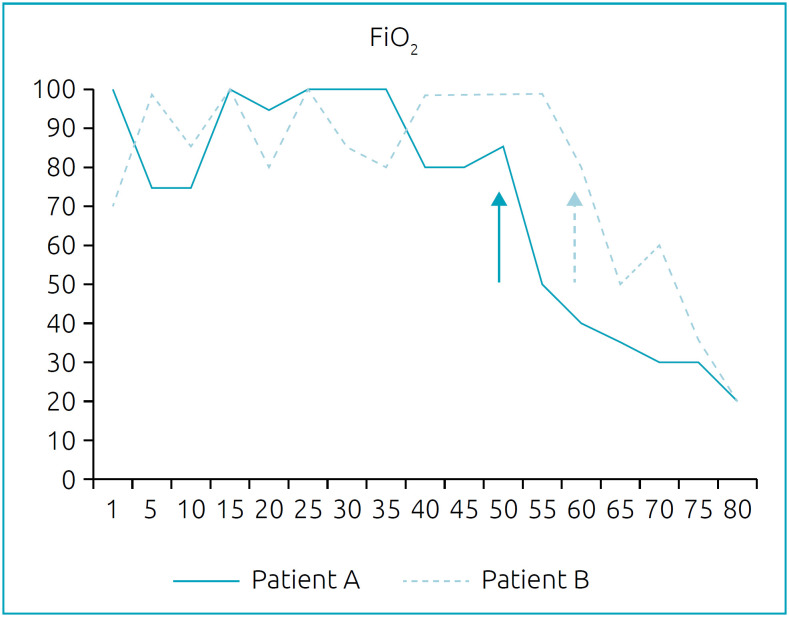
Fraction of inspired oxygen of patients A and B during the Pediatric Intensive Care Unit stay. Arrows indicate the start of dornase alfa administration.

Figure 3 shows the need for FiO_2_ of patients A and B during the PICU stay. The decline since the initiation of rhDNase treatment is noteworthy (day 50 for patient A and day 60 for patient B).

## DISCUSSION

VILI is a frequent complication observed in prolonged mechanical ventilated children with respiratory infections. In the patients analyzed in this study, lung collapse/atelectasis was observed in both cases. The incidence of this complication is estimated in 8–54% of pediatric patients on mechanical ventilation, with a concomitant increase in the morbidity and length of stay. Difficulties in lung clearance, in addition to the reduction of functional residual capacity in this type of patients, may explain atelectasis in these patients.^8^ ^,^ ^9^

In patients with lung infections complicated by atelectasis, respiratory secretions and mucus have a high concentration of DNA. DNA has an intrinsic tendency to form a viscous gel, with increasing viscosity and adhesiveness of the mucus.^10^ For this reason, rhDNase may be an effective treatment in these patients.

Despite the fact that rhDNase treatment was daily administered to only two patients, the present analytic case series suggests that it can be a valid therapeutic choice for these patients. Indeed, it may be associated to a quicker resolution of atelectasis and, therefore, to a possible shorter mechanical ventilation duration and to a shorter length of stay in PICU. If the mechanical ventilation period is minimized, the comorbidity and all the risks associated to this procedure can be reduced.

There are several studies published in the literature on the rhDNAse administration to non-CF patients: case reports,^11^ ^–^ ^14^ retrospective studies,^6^ ^,^ ^8^ a classic review,^15^ systematic reviews,^1^ ^,^ ^16^ a non-randomized controlled trial^7^ and a randomized controlled trial.^4^ Most of them suggest a clinical improvement after treatment with rhDNAse in patients with atelectasis.

Riethmueller et al. showed some advantages in rhDNase use in pediatric patients such as an improvement in atelectasis with shorter mechanical ventilation duration and ICU length of stay.^4^ ^,^ ^5^ Prodhan et al., in 2009, also showed improvements in radiologic chest x-ray scores of atelectasis.^8^ Other studies conducted with smaller samples showed the same trend for rhDNAse treatment, such as Fedakar et al., in 2012, in neonatal patients^7^ and Hendriks et al., in 2005.^6^

Some studies had inconsistent results with rhDNAse treatment, which is why some authors, such as Papacostas and Strickland, stated that the routine use of rhDNase in non-CF population cannot be recommended.^15^ ^,^ ^16^ However, some prior studies showed an earlier extubation in patients treated with dornase alfa.

The intratracheal administration seemed to be safe in the present clinical setting. In these two patients, the effects of rhDNase were apparently similar to that verified in CF patients, with improvement in viscous respiratory secretions. Patient B was 17 months of age at the time of admission, which is less than the usual age in CF patients (above five years of age).

In both patients, since the beginning of rhDNase administration, the FiO_2_ should decrease as well as the PaO_2_/FiO_2_ ratio and the PaCO_2_. A decreasing trend in respiratory rate and peak inspiratory pressure was found in these patients.

The chest x-ray was evaluated according to a score system previously used in other study, which defined a consistent classification with good agreement between the experts reading the chest radiographs.^6^ There was a strong agreement on the favourable radiologic course among both patients described in this study (see images).

There were several limitations in the interpretation and exploratory analysis of these two clinical cases. First, the main limitation was related to the retrospective nature; second, only two patients in a specific clinical setting were presented, making it difficult to generalize the results; third, the clinical course was very unpredictable and different in the two cases. Nevertheless, no difference was found between the rhDNase technical procedure in both patients. Other limitation was the classification of the patients’ chest radiographs, but the authors sought to overcome this issue by having two independent experienced intensivists to interpret the x-ray findings.

No major complications, such as immediate oxygen desaturation, pulmonary hemorrhage or worsening of pulmonary parameters associated with dornase alfa therapy, were found in the study patients.

In conclusion, rhDNase use is emphasized as a helpful tool to intubated patients admitted to PICU, with high mechanical ventilation settings and atelectasis. Therefore, further studies are needed to assess and, possible, to propose valid indications for the use of rhDNase in this type of patients.
